# Supplementary material for: A Quadruplex RT-qPCR for the Detection of Porcine Sapelovirus, Porcine Kobuvirus, Porcine Teschovirus, and Porcine Enterovirus G
Source: Animals (Basel). 2025 Mar 31;15(7):1008. doi: 10.3390/ani15071008 (PMC11987865; doi:10.3390/ani15071008)
Supplement: Supplementary file 1 [file animals-15-01008-s001.zip › animals-3477552-supplementary.pdf]

Supplementary Materials

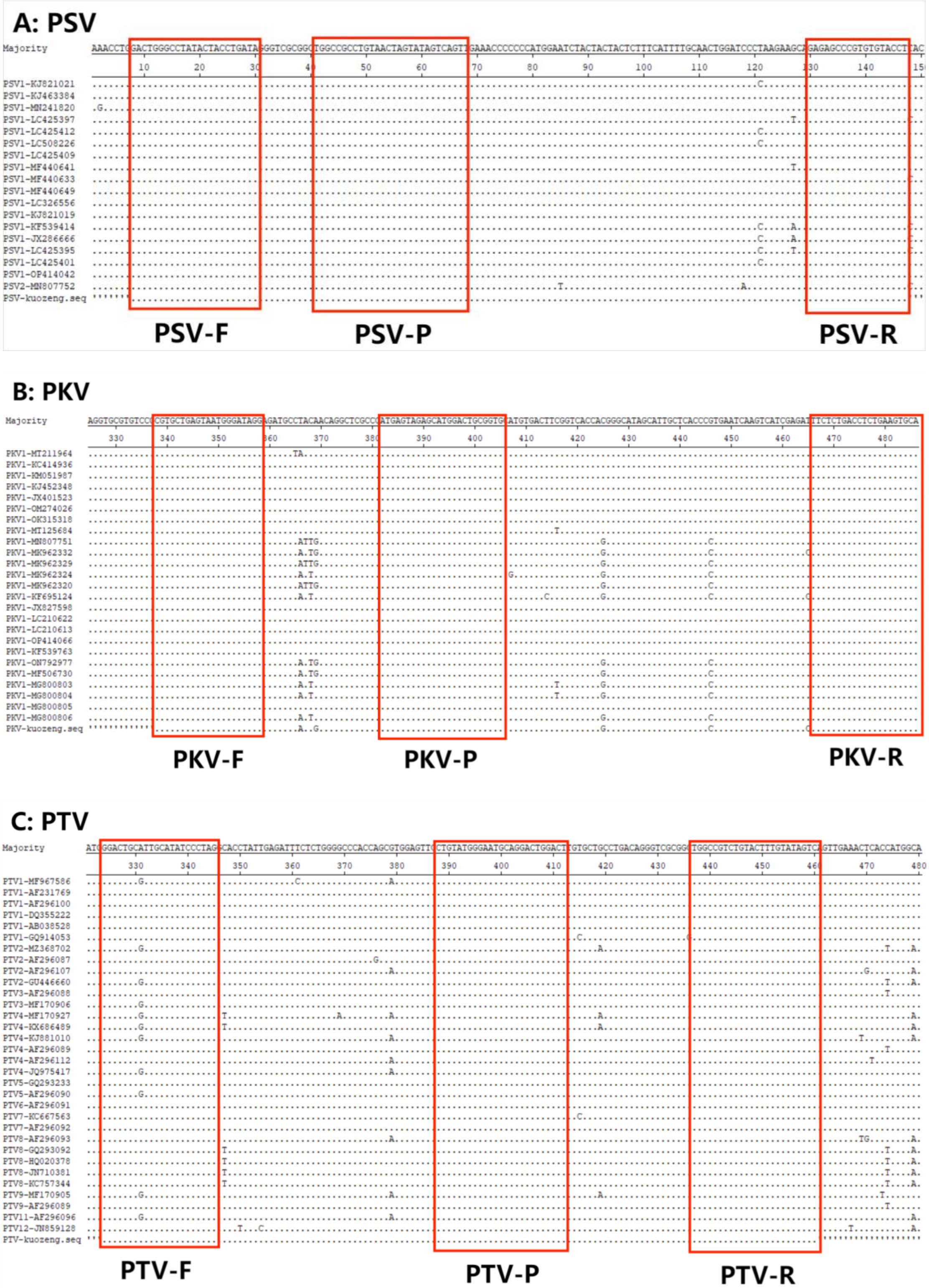

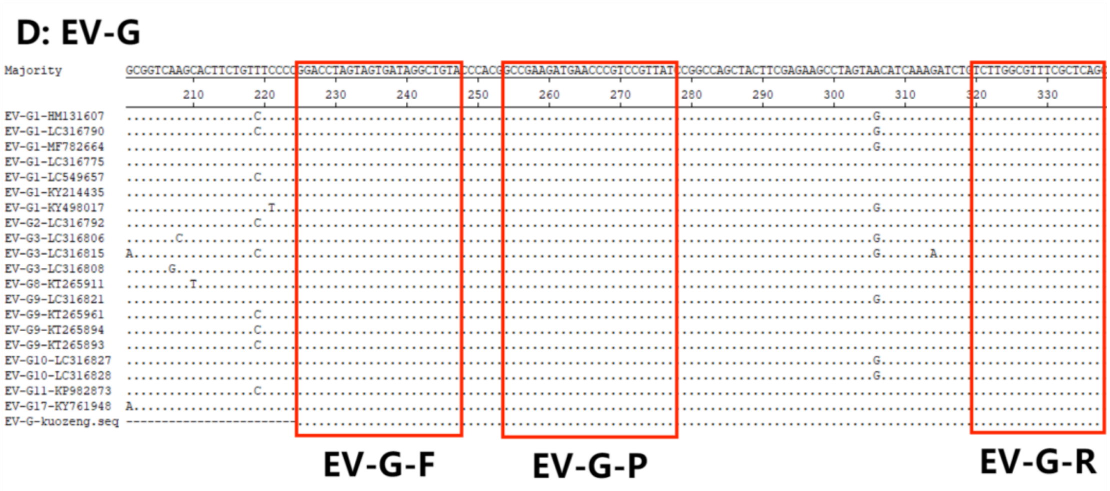

Figure S1. The multiple sequence alignments of PSV (A), PKV (B), PTV (C), and EV-G (D). The forward (F)/reverse (R) primers, and probe (P) are located in the conserved region of 5' UTR.

Table S1. Agreements of the test results via different assays.

Method	Positive Samples			
	PSV	PKV	PTV	EV-G
The Developed Quadruplex RT-qPCR	15.25% (278/1823)	21.72% (396/1823)	18.82% (343/1823)	27.10% (494/1823)
The Reported Reference RT-qPCR	15.03% (274/1823)	21.56% (393/1823)	18.65% (340/1823)	26.80% (488/1823)
Agreements (95% CI)	99.56% (99.14-99.78%)	99.40% (98.92-99.66%)	99.51% (99.06-99.74%)	99.01% (98.44-99.37%)
